# THE DEVELOPMENT OF ADHERENCE SUPPORT MESSAGES FOR AN AI-BASED REMINDER SYSTEM

**DOI:** 10.1093/geroni/igac059.282

**Published:** 2022-12-20

**Authors:** Shenghao Zhang, Michael Dieciuc, Andrew Dilanchian, Walter Boot

**Affiliations:** Florida State University, Tallahassee, Florida, United States; Florida State University, Tallahassee, Florida, United States; Florida State University, Tallahassee, Florida, United States; Florida State University, Tallahassee, Florida, United States

## Abstract

Artificial Intelligence (AI) holds tremendous potential for aiding in the customization and tailoring of interventions. This talk will focus on the initial development of an adherence support system to maintain older adults' engagement with at-home cognitive training and assessment delivered via mobile devices. As part of the NIA funded Adherence Promotion with Person-centered Technology (APPT) project, a reminder and adherence support system is being developed to automatically detect adherence lapses and provide just-in-time reminders with tailored messaging to facilitate reengagement. The results of an initial study (N=40) will be presented in which participants were delivered reminders via SMS text messaging that were either tailored or not tailored to their self-reported motivation to engage with the intervention. Tailored messages were perceived as more motivating, and qualitative data informed additional ways in which messages might further be tailored based on individual preferences to support adherence.

